# Ethical considerations in public engagement: developing tools for assessing the boundaries of research and involvement

**DOI:** 10.1186/s40900-024-00617-8

**Published:** 2024-08-07

**Authors:** Jaime Garcia-Iglesias, Iona Beange, Donald Davidson, Suzanne Goopy, Huayi Huang, Fiona Murray, Carol Porteous, Elizabeth Stevenson, Sinead Rhodes, Faye Watson, Sue Fletcher-Watson

**Affiliations:** 1 Centre for Biomedicine, Self and Society, Usher Institute, University of Edinburgh, Edinburgh, UK; 2 Centre for Inflammation Research, Institute for Regeneration and Repair, University of Edinburgh, Edinburgh, UK; 3 Usher Institute, University of Edinburgh, Edinburgh, UK; 4 School of Health in Social Science, Medical School, University of Edinburgh, Edinburgh, UK; 5 Edinburgh Clinical Research Facility, University of Edinburgh, Edinburgh, UK; 6 Biomedical Teaching Organisation, University of Edinburgh, Edinburgh, UK; 7 Centre for Clinical Brain Sciences, University of Edinburgh, Edinburgh, UK; 8 College for Medicine and Veterinary Medicine, University of Edinburgh, Edinburgh, UK

**Keywords:** Public engagement, Ethical approval, Ethical review, Power, Responsibility, Managing risks, Patient-oriented research, Patient-centred research, And patient engagement

## Abstract

Public engagement with research (PEwR) has become increasingly integral to research practices. This paper explores the process and outcomes of a collaborative effort to address the ethical implications of PEwR activities and develop tools to navigate them within the context of a University Medical School. The activities this paper reflects on aimed to establish boundaries between research data collection and PEwR activities, support colleagues in identifying the ethical considerations relevant to their planned activities, and build confidence and capacity among staff to conduct PEwR projects. The development process involved the creation of a taxonomy outlining key terms used in PEwR work, a self-assessment tool to evaluate the need for formal ethical review, and a code of conduct for ethical PEwR. These tools were refined through iterative discussions and feedback from stakeholders, resulting in practical guidance for researchers navigating the ethical complexities of PEwR. Additionally, reflective prompts were developed to guide researchers in planning and conducting engagement activities, addressing a crucial aspect often overlooked in formal ethical review processes. The paper reflects on the broader regulatory landscape and the limitations of existing approval and governance processes, and prompts critical reflection on the compatibility of formal approval processes with the ethos of PEwR. Overall, the paper offers insights and practical guidance for researchers and institutions grappling with ethical considerations in PEwR, contributing to the ongoing conversation surrounding responsible research practices.

**Supplementary Information:**

The online version contains supplementary material available at 10.1186/s40900-024-00617-8.

## Abstract (plain language summary)

This paper talks about making research fairer for everyone involved. Sometimes, researchers ask members of the public for advice, guidance or insight, or for help to design or do research, this is sometimes known as ‘public engagement with research’. But figuring out how to do this in a fair and respectful way can be tricky. In this paper, we discuss how we tried to make some helpful tools. These tools help researchers decide if they need to get formal permission, known as ethical approval, for their work when they are engaging with members of the public or communities. They also give tips on how to do the work in a good and fair way. We produced three main tools. One helps people understand the important words used in this kind of work (known as a taxonomy). Another tool helps researchers decide if they need to ask for special permission (a self-assessment tool). And the last tool gives guidelines on how to do the work in a respectful way (a code of conduct). These tools are meant to help researchers do their work better and treat everyone involved fairly. The paper also talks about how more work is needed in the area, but these tools are a good start to making research fairer and more respectful for everyone.

**Supplementary Information:**

The online version contains supplementary material available at 10.1186/s40900-024-00617-8.

## Introduction

In recent decades, “public involvement in research” has experienced significant development, becoming an essential element of the research landscape. In fact, it has been argued, public involvement may make research better and more relevant [7, p. 1]. Patients’ roles, traditionally study participants, have transformed to become “active partners and co-designers” [17, p. 1]. This evolution has led to the appearance of a multitude of definitions and terms to refer to these activities. In the UK, the National Co-ordinating Centre for Public Engagement, defines public engagement as the “many ways organisations seek to involve the public in their work” [9]. In this paper, we also refer to “public involvement,” which is defined as “research being carried out ‘with’ or ‘by’ members of the public rather than ‘to’, ‘about’ or ‘for’ them” (UK Standards for Public Involvement). Further to this, the Health Research Authority (also in the UK), defines public engagement with research as “all the ways in which the research community works together with people including patients, carers, advocates, service users and members of the community” [6]; [9]. These terms encompass a wide variety of theorizations, levels of engagement, and terminology, such as ‘patient-oriented research’, ‘participatory’ research or services or ‘patient engagement’ [17, p. 2]. For this paper, we use the term ‘public engagement with research’ or PEwR in this way.

Institutions have been set up to support PEwR activities. In the UK these include the UK Standards for Public Involvement in Research (supported by the National Institute for Health and Care Research), INVOLVE, and the National Coordinating Centre for Public Engagement (NCCPE). Most recently, in 2023, the UK’s largest funders and healthcare bodies signed a joint statement “to improve the extent and quality of public involvement across the sector so that it is consistently excellent” [6]. In turn, this has often translated to public engagement becoming a requisite for securing research funding or institutional ethical permissions [3, p. 2], as well as reporting and publishing research [15]. Despite this welcomed infrastructure to support PEwR, there remain gaps in knowledge and standards in the delivery of PEwR. One such gap concerns the extent to which PEwR should be subject to formal ethical review in the same way as data collection for research.

In 2016, the UK Health Research Authority and INVOLVE published a joint statement suggesting that “involving the public in the design and development of research does not generally raise any ethical concerns” [7, p. 2]. We presume that this statement is using the phrase ‘ethical concerns’ to narrowly refer to the kinds of concerns addressed by a formal research ethics review process, such as safeguarding, withdrawal from research, etc.^1^. To such an extent, we agree that public involvement with research is not inherently ‘riskier’ than other research activities.

Furthermore, a blanket need for formal ethical review risks demoting or disempowering non-academic contributors from the roles of consultants, co-researchers, or advisors to a more passive status as participants. Attending a meeting as an expert, discussing new project ideas, setting priorities, designing studies and, or interpreting findings does not require that we sign a consent form. Indeed, to do so clearly removes the locus of power away from the person signing and into the hands of the person who wrote the consent form. This particular risk is exacerbated when institutional, formal ethical review processes operate in complex, convoluted and obscure ways that often baffle researchers let alone members of the public.

However, we also recognize that PEwR is not without potential to do harm – something which formal research ethics review aims to anticipate and minimise. For example, a public lecture or a workshop could cause distress to audience members or participants if they learn for the first time that aspects of their lifestyle or personal history put them at higher risk of dementia. When patients are invited to join advisory panels, they may feel pressure to reveal personal details about their medical history to reinforce their expertise or legitimise their presence – especially in a room where most other people have potentially intimidating professional qualifications. Some patient groups may be exploited, if research involvement roles are positioned as an opportunity, or even a duty, and not properly reimbursed. When patients are more deeply involved in research roles, such as collecting or analysing data, they might experience distress, particularly if interacting with participants triggers their own painful or emotional memories [14, p. 98]. Thus, at all levels of PEwR from science communication to embedded co-production, there is a danger of harm to patients or members of the public, and a duty of care on the part of the research team and broader institution who invited them in.

These concerns are not accessory to PEwR activities but rather exist at their heart. Following a review on the impacts of public engagement, Brett et al. conclude that “developing a wide view which considers the impact of PPI [public and patient involvement] on the people involved in the process can be critical to our understanding of why some studies that involve patients and the public thrive, while others fail” [1, p. 388]. Despite the importance of these considerations, there is a stark absence of consistent guidance as to whether different forms of PEwR require formal ethical review. Nor is there, to our knowledge, any sustained attempt to provide a framework for ethical conduct of PEwR in the absence of formal review (see Pandya-Wood et al. [11]; Greenhalgh et al. [5]). This is, in part, due to there being a wide heterogeneity of practices, communities, and levels of engagement [8, p. 6] that resists generalizable principles or frameworks.

The lack of frameworks about whether or how PEwR requires formal ethical review can, ironically, be a key barrier to PEwR happening. In our work as members of a university ethics review committee, we have found this lack of guidance to hamper appropriate ethical PEwR in several ways. Researchers may avoid developing PEwR initiatives altogether for fear of having to spend time or resources in securing formal ethical review (especially when this process is lengthy or resource-intensive). Likewise, they may avoid PEwR for fear that its conduction would be unethical. On the other hand, others could assume that the lack of a requirement for formal ethical review means there are no ethical issues or risks involved in PEwR.

Similarly, experts in PEwR who are not experienced with formal research ethics review may face barriers as their PEwR process becomes more elaborate, in-depth, or complex. For example, although a priority-setting exercise with members of an online community of people with depression was assessed as not requiring ethics review, the funding panel requested that formal ethics review be undergone for a follow-up exercised aimed at collecting data answering one of the priority questions identified in the previous priority-setting. It is crucial that innovations in PEwR and findings from this work are shared and yet academic teams may be unable to publish their work in certain journals which require evidence of having undergone formal ethical review. Finally, ethics committees such as ours often must rely on anecdotal knowledge to make judgements about what does or does not require formal ethical review, given the absence of standardized frameworks.

### About this paper

In this paper, we report and reflect on the development of specific tools and processes for assessing the ethical needs of PEwR initiatives, as members of an ethics review committee for a large University medical school. These tools aim to delineate boundaries between research data collection and PEwR activities of various kinds, provide a self-assessment framework for ethical practice in PEwR and, overall, give people greater confidence when conducting PEwR work. We describe and critically reflect on the development of the following resources:

1. a taxonomy to define key terms relating to PEwR with associated resource recommendations.
2. a self-assessment tool to support people understanding where their planned activities fall in relation to research or PEwR.
3. a code of conduct for ethical conduct of PEwR (appended to the self-assessment tool).

We will, first, describe our work as part of an institutional ethics committee, the identification of a need for specific guidance, and our key assumptions; we will then describe the process of developing these tools and processes; provide an overview of the tools themselves; and reflect on early feedback received and future work needed.

## Developing specific tools for PEWR in ethics

### Identifying needs, goals and outputs

The Edinburgh Medical School Research Ethics Committee (EMREC) provides ethical opinions to members of staff and postgraduate researchers within the University of Edinburgh Medical School in relation to planned research to be conducted on humans i.e. their data, or tissues. These research activities come from a wide range of disciplines, including public health, epidemiology, social science or psychology. EMREC does not review research that involves recruitment of NHS patients, use of NHS data, or other health service resources: such projects are evaluated by an NHS research ethics committee. EMREC is led by two co-directors and formed of over 38 members, which include experienced academics and academic-clinicians from a variety of disciplines. There are also 2–4 lay members who are not researchers.

EMREC receives regular enquiries about whether a specific piece of PEwR work (such as holding a workshop with people living with endometriosis to identify research priorities or interviewing HIV activists about their work during COVID-19) requires formal ethics review. In addition, often teams contact EMREC following completion of a PEwR activity that they want to publish because the journal in which they wish to publish has requested evidence of the work having undergone formal ethics approval. These enquiries are happening in the context of an institutional investment in staffing, leading to a significant degree of distributed expertise across the Medical School about diverse forms of PEwR.

Responding to this, in the summer of 2022, a Public and Patient Involvement and Engagement working group was formed by EMREC with the aim of developing new tools and processes to navigate the ethical implications of PEWR within the University of Edinburgh Medical School. The group’s original understandings were that:

1. PEwR is both important and skilled work that presents a unique set of ethical implications,
2. PEwR is a fragmented landscape where many people have relevant but different expertise and where a wide range of terminology is in use, and.
3. there is no existing widely-agreed framework for ethical PEwR.

This working group was designed to be temporary, lasting approximately six months. It was composed of eleven members with different degrees of seniority and disciplinary backgrounds - both members of EMREC and those from other parts of the Medical School, and other parts of the University of Edinburgh. Among these, there were both academics and PEwR experts in professional services (i.e. primarily non-academic) roles. The working group met four times (August, September and November 2022; and January 2023).

The group identified three key goals and, in relation to these, key outputs needed. The goals were: (1) help establish boundaries between research data collection (requiring an ethical opinion from EMREC) and PEwR activities of various kinds (requiring ethical reflection/practice but not a formal EMREC ethical opinion), (2) support colleagues to identify where their planned activities fell in the research-PEwR continuum and consequently the relevant ethical framework, and (3) identify ways of building confidence and capacity among staff to conduct PEwR projects. In relation to these goals, the working group initially agreed on producing the following key outputs:

- A taxonomy outlining and defining key terms used in the PEwR work, with examples. While not universal or definitive, the taxonomy should help colleagues identify and label their activities and help determine the ethical considerations that would apply to conduct the work with integrity. It would also facilitate conversations between staff with varying levels and types of experience, and ensure that decisions around ethical conduct would be based on more than choice of terminology.
- A self-assessment tool to provide a more systematic way to evaluate whether a given academic activity, involving a non-academic partner (organisation or individual) requires formal evaluation by a research ethics committee.
- A list of resources collected both from within and beyond our institution that are relevant to the issue of ethics and PEwR and can serve as ‘further reading’ and training.

While we aimed to develop this work with a view to it being useful within the remit of the University of Edinburgh Medical School, we also understood that there was significant potential for these outputs to be of interest and relevance more widely. In this way, we aimed to position them as a pragmatic addition to existing guidance and resources, such as the NIHR Reflective Questions [2].

## Our process

Across the first three meetings, the group worked together on the simultaneous development of the three outputs (taxonomy, self-assessment tool, and resources). The initial taxonomy was informed by the guidance produced by the Public Involvement Resource Hub at Imperial College London [10]. The taxonomy was developed as a table that included key terms (such as ‘public engagement’, ‘co-production’, or ‘market research’), with their definitions, examples, and synonyms. From early on, it was decided that different key terms would not be defined by the methods used, as there could be significant overlap among these – e.g. something called a *focus group* might be a part of a consultation, market research or research data collection.

A draft table (with just six categories) was presented in the first meeting and group members were asked to work on the table between meetings, including providing additional examples, amending language, or any other suggestions. This was done on a shared document using ‘comments’ so that contradictory views could be identified and agreements reached. The table was also shared with colleagues from outside the University of Edinburgh Medical School to capture the range of terminologies used across disciplines, recognising the interdisciplinary nature of much research.

Through this process, additional key terms were identified, such as “science communication” and “action research,” definitions were developed more fully, and synonyms were sometimes contextualized (by indicating, for example, shades of difference or usages specific to an area). Upon further work, three additional sections were added to the taxonomy tool: first, an introduction was developed that explained what terminology our specific institution used and noted that the boundaries between different terms were often “fuzzy and flexible.” In addition, the group agreed that it would be useful to provide a narrative example of how different forms of public engagement with research might co-exist and flow from one to another. To this end, a fictional example was developed where a team of clinical researchers interested in diabetes are described engaging in scoping work, research, co-production, science communication and action research at different times of their research programme. Finally, a section was also added that prompted researchers to reflect on the processes of negotiating how partners can be described in research (for example, whether to use terms such as ‘patient’ or ‘lay member’).

For the self-assessment tool, a first iteration was a table with two columns (one for research or work requiring formal ethical review and one for PEwR or work not requiring formal ethical review). The aim was for group members to fill the table with examples of activities that would fall under each category, with a view to identifying generalizable characteristics. However, this task proved complicated given the wide diversity of possible activities, multitude of contexts, and sheer number of exceptions. To address this, group members were asked to complete a case-based exercise. They were presented with the following situation: “I tell you I’m planning a focus group with some autistic folk” and asked how they would determine whether the activity would be a form of data collection for a research project (requiring formal ethical review) or another form of PEwR. Group members were asked, with a view to developing the self-assessment tool, to identify which questions they would ask to assess the activity. The replies of working group members were synthesized by one of the authors (SFW) and presented at the following meeting.

Through discussion as a group, we determined that the questions identified as useful in identifying if an activity required formal ethical review fell, roughly, under four main areas. Under each area, some indicators of activities were provided which were “less likely to need ethics review” and some “more likely to need ethics review”. The four umbrella questions were:

1. What is the purpose and the planned outcome of the activity? (see Table 1 for an excerpt of the initial draft answer to this question)
2. What is the status of the people involved in the activity? (indicators of less likely to need ethics review were “participants will be equal partners with academic team” or “participants will be advisors” and indicators more likely to require ethics approval were “participants will undertake tasks determined by academics” or “participants will contribute data or sign consent forms”).
3. What kind of information is being collected? (indicators of less likely to need ethics review were “asking about expert opinion on a topic” or “sessions will be minuted and notes taken” and indicators more likely to require ethics approval were “sessions will be recorded and transcribed” or “asking about participants’ personal experiences”).
4. What are the risks inherent in this activity? (indicators of less likely to need ethics review were “participants will be involved in decision-making” or “participants will be credited for their role in a manner of their choosing” and indicators more likely to require ethics approval were “participants’ involvement will and must be anonymized fully” or “participants have a choice between following protocol or withdrawing from the study”).

**Table 1:** Excerpt from the first version of the self-assessment tool showing some of the indicative activities that were identified less or more likely to need ‘research ethics review’

What is the purpose and the planned outcome of the activity?	
Less likely to need research ethics review	More likely to need research ethics review
To plan a new research project	To answer a specific research question
To develop a list of research questions	To describe community members’ attitudes to, opinions on, or experience of research or practice
Information gathered will be used to help write a grant	Information gathered will be used to write a journal paper

Upon further work, the group decided to modify this initial iteration in several ways leading to the final version. First, a brief introduction explaining the purpose of the tool was written. This included information about the aims of the tool, and a very brief overview of the process of formal research ethics review. It also emphasised the importance of discussion of the tool within the team, with PEwR experts and sometimes with EMREC members, depending on how clear-cut the outcome was. Second, we included brief information about what are ‘research’ and ‘public engagement with research’ with a view to supporting people who may not be familiar with how these concepts are used by ethics review committees (for example, lay co-applicants or co-researchers). Third, we included key guidance about how to use the tool, including ‘next steps’ if the activity was determined to be research or engagement. Importantly, this emphasised that none of the questions posed and indicators given were definitive of something needing or not needing formal research ethics review, but instead they should be used collectively to signpost a team towards, or away from, formal review.

Finally, while the four umbrella questions remained the same as in the previous iteration, the indicators under each were further refined. In discussing the previous version, the group agreed that, while some indicators could relate to an activity falling into either category (research or engagement) depending on other factors, there were others that were much more likely to fall under one category than the other. In other words, while no single indicator was deterministic of needing or not needing formal review, some indicators were more influential than others on the final self-assessment outcome. Thus, we divided the indicators associated with each umbrella question into two sub-groups. The more influential indicators were labelled as either “probably doesn’t need ethical review” or “almost certainly needs ethical review”. Less influential indicators were labelled as either “less likely to need ethical review” or “more likely to need ethical review.” This is shown in Table 2.

**Table 2:** Excerpt from final version of the taxonomy, showing four groupings of indicators

What is the purpose and the planned outcome of the activity?	
Probably doesn’t need formal ethics review	Almost certainly needs formal ethics review
To plan a new project or activity	To answer a specific research question
Main goal is relationship-building and insight	Main goal is systematic generation of knowledge
Less likely to need formal ethics review	More likely to need formal ethics review
To develop a list of research questions or priorities	To formally synthesise and describe attitudes to, opinions on, or experience of research or practice
Information gathered will be used to help write a grant or to co-create public-facing materials such as a web page, flyer or video	Information gathered will be used to write a journal paper

This new format retains the awareness of the sometimes-blurry lines between research and PEwR for many activities, but also seeks to provide stronger direction through indicative activities that are more clear-cut, with a particular view to supporting early-career researchers and people new to ethics reviews and/or engagement processes.

A key concern of the group was what would happen next if a planned activity, using the self-assessment tool, was deemed as PEwR. The formal review process for research would not be available for a planned activity identified as PEwR i.e. completing a series of documents and a number of protocols to deal with issues such as data protection, safeguarding, etc. This would leave a vacuum in terms of guidance for ethical conduction of PEwR. The group was concerned that some people using the self-assessment tool might arrive at the conclusion that their planned activity was entirely without ethical risks, given that it was not required to undergo formal review. Others might be conscious of the risks but feel adrift as to how to proceed. This was a particular concern with early-career researchers and indeed established academics turning to PEwR for the first time: we wanted to facilitate their involvement with PEwR but we were also aware that many may lack experience and resources. To address this, the group decided to develop an additional output comprising a series of reflective prompts to guide researchers in planning and conducting engagement activities.

The prompts were organized under four headings. First, “Data Minimisation and Security” included information about required compliance with data protection legislation, suggestions about collecting and processing information, and ideas around ensuring confidentiality. Second, “Safeguarding Collaborators and Emotional Labour” prompted researchers to think about the risk of partners becoming distressed and suggested what things should be planned for in this regard. Third, “Professional Conduct and Intellectual Property” included advice on how to clearly manage partners’ expectations around their contributions, impact, and intellectual property. Finally, fourth, under “Power Imbalances”, the guidance discusses how researchers may work to address the inherent imbalances that exist in relationships with partners. It prompts the researcher to think about choice of location, information sharing, and authorship among others. While the Edinburgh Medical School Research Ethics Committee remains available for consultation on all these matters, as well as dedicated and professional PEwR staff, the group developed these guidelines with a view both to emphasizing the fact that an activity not requiring formal ethical review did not mean that the activity was absent of risk or did not require careful ethical planning; and to support those who may be unfamiliar with how to develop engagement activities. It was decided that this guideline should follow the self-assessment tool for clarity.

Finally, in the process of developing these outputs (the planned taxonomy and assessment tool, and the additional reflective prompts appended to the assessment tool), the group collected a large number of resources, including academic papers (e.g. Staniszewska et al. [16]; Schroeder et al. [13]; Redman et al. [12]; Fletcher-Watson et al. [4]), guidance produced by other institutions, and key online sites with information about national frameworks or policy. Among these, key resources were selected and appended to the taxonomy document. The final version of these documents can be found as appendices (Supplementary Material 1: Assessment tool and reflective prompts; Supplementary Material 2: Taxonomy and resources).

## Further considerations and early results

The guidance and tools presented here are designed to clarify a boundary between research and engagement that is poorly defined and could cause harm if not well understood. In sharing them, we aim to facilitate researchers’ engagement with PEwR by providing familiarity with the terminology and approaches, examples, and suggesting key considerations. Most importantly, they support researchers to determine whether their planned activity should undergo a formal ethical review process or not – and if not, guides them towards ethical conduct in the absence of formal review. Reflecting on the process much of what we have explained essentially reflects a distinction between PEwR and research data collection that can be encapsulated within the idea of ‘locus of control’: namely that during PEwR the locus of control, as far as possible, sits with the engaged communities or members.

It should be noted, however, that researchers and these guidance and tools exist within a larger landscape, with added regulatory processes. Thus, researchers may need (regardless of whether their planned activity is research or engagement) to navigate additional compliance such as data protection or information security protocols and / or to consider reputational risk associated with certain topics. We are aware that the overlap of complex and sometimes obscure regulatory demands complicates the task of conducting both research and PEwR, as it requires researchers to juggle multiple procedures, documents, and approvals. This publication does not resolve all the questions that exist, but it does attempt to take a bold step towards confronting grey areas and providing systematic processes to navigate them.

The outputs described above were made available on the University of Edinburgh Medical School Research Ethics Committee intranet site under the heading “Public Engagement with Research.” While we do not collect statistics on the number of times the resources have been used, the committee has received positive feedback from people who have engaged with the documents. For example, one researcher commented that, in the process of developing an engagement activity, they had been “grappling with precisely these questions (of whether this qualifies as research, and whether it requires ethical review)” and that the documents were “quite timely and helpful. It allows me to think about these considerations in a systematic manner and it’s handy for me to send on to others as a framework for discussion should we have differing opinions.” It was this mention to the possibility of these documents being used as a framework for discussion that prompted us to write this paper as a way of sharing them beyond the University of Edinburgh College of Medicine and Veterinary Medicine (where they are already used for training early-career researchers and in the MSc in Science Communication and Public Engagement). While we think they can be useful, we also encourage potential users to adapt them to their specific contexts, with different institutions potentially establishing differing procedures or requirements. To that end, we have shared in this paper the process of writing these documents so that other people and teams may also think through them productively and creatively.

## Final reflections

In developing these documents, we sought to answer a need among members of our immediate community, seeking to better assess whether an activity required formal ethical review and wanting guidance to ethically conduct PEwR work. However, we also came to realize the limitations of existing approval and governance processes. In our case, a key reason why these documents were developed is because existing formal ethical review processes would not be adequate to capture the particularities and complexities of PEwR in our large, diverse Medical School.

Looking back at the tools we developed and the feedback received, we are also satisfied with the pragmatic approach we took. There is a vast amount of resources and literature available about how to conduct PEwR, as well as a multitude of accounts and reflections both of an anecdotal and epistemological nature. Building on this conceptual work and associated principles, we sought to develop pragmatic, clear, applicable tools, without overwhelming users with a multitude of available resources and complex theory. This is, we feel, particularly applicable to contexts like ours: a large, very diverse medical school which encompasses biomedical to social science disciplines where researchers and funders have vastly differing expectations and knowledge of PEwR.

This process also led us to reflect on the practical functions of formal ethical review. Formal ethics approval provides applicants with structured resources to think and plan about their work, feedback and guidance about their plans, and—most commonly—a code and letter than can be used to easily report to journals that your research has met a specific ethical threshold. With these documents we have sought to provide some similar, pragmatic guidance to support and empower people, through a self-assessment process. This begs the question, what, if any, formal approval processes should be developed for PEwR? Are such formal processes in any way adequate to the ethos of PEwR? Would formal independent review necessarily conflict with the values of PEwR, namely the empowerment of community members as decision-makers and experts? Thus, these documents and this paper contribute to an ongoing conversation as PEwR continues to develop in frequency and sophistication in health and social care research.

### Electronic supplementary material

Below is the link to the electronic supplementary material.

Supplementary Material 1

Supplementary Material 2

## Data Availability

No datasets were generated or analysed during the current study.

## Footnotes

1 The difference between research and public engagement is a complex one. Formal ethics approval, which is often seen as a regulatory or compliance mechanism, may not always be a good marker of this boundary, as it may ignore complex issues such as the distribution of power, the ethos of the activities, or their aims. Furthermore, different institutions use different criteria to determine what activities require ethics approval or are considered research. In this paper we reflect on the process of developing tools which we intended as pragmatic interventions that would support researchers, especially those without previous experience of PEwR to label their planned activities and understand their implications. Thus, we employ—even if not at all times comfortably—the framework that equates research with activities requiring ethics approval and PEwR with activities not requiring ethics approval.
