# Supplementary material for: Ethical considerations in public engagement: developing tools for assessing the boundaries of research and involvement
Source: Res Involv Engagem. 2024 Aug 7;10:83. doi: 10.1186/s40900-024-00617-8 (PMC11305016; doi:10.1186/s40900-024-00617-8)
Supplement: Supplementary file 1 — Supplementary Material 1 [file 40900_2024_617_MOESM1_ESM.pdf]

KEY TERMS IN PUBLIC ENGAGEMENT WITH RESEARCH
CMVM public engagement & ethics working group

Why do we need this table?
Public engagement means different things to different people. While our college CMVM uses "Public Engagement with Research" to cover everything from simple dissemination of science, through to co-production, this breadth makes it quite hard for people to navigate the space. Likewise "Knowledge Exchange" is used for work with the public but also industry and government. Clarifying terminology for particular forms of public engagement with research can help us determine the ethical considerations that apply in each case, and conduct the work with integrity. The definitions used here are not universal but are intended to be useful as a reference for EMREC and for other research ethics committees within CMVM. They may be especially useful for novices. The boundaries between the categories described below are both fuzzy and flexible.

Key term	Definition	Examples	Synonyms?
Science Communication	Knowledge about how research is done, is shared with the public / specific groups.<br>Information about new research findings are shared with the public in an accessible way.<br>Communication may be one way (from researcher to public) or include dialogue / feedback.	Science Festival event<br>Public lecture<br>Social media feed<br><br>Any of these may include some feedback or response options	Dissemination<br>Outreach<br>*Internationally, the term "science communication" often covers a broader spectrum of activity from dissemination to dialogue about science.*
Public Involvement	Members of the public / specific groups influence and shape what and how research is done.<br>In CMVM research, these groups are often patients, service users or their carers / family.	Clinical trial advisory board<br>Priority-setting exercise<br>Opinion polling (e.g. at events, on social media) on research planning<br>Consultation exercise, e.g. on study design	Participatory or Inclusive Research<br>CBPR (community-based participatory research)<br>Consultation<br>User Testing<br>PPI (Public & Patient Involvement)<br>Community Engagement
Co-Production	Members of the public / specific groups have research leadership roles<br>Non-academics (e.g. patients) working alongside academics as equal partners<br>Often involves creation of something new, where public and practitioners share ownership in its development	Research / charity partnership<br>Practitioner / PPI or community partnership<br>Co-creation of an information video for patients, an outcome measure, an intervention, or a new diagnostic / treatment pathway	Participatory research<br>Partnership or Collaboration<br>Co-creation<br>Co-design (where community input is largely limited to design, not delivery)<br>Emancipatory research (where community partners gain skills or opportunities)
Citizen Science	Members of public involved in collection and analysis of massive data sets<br>OR, more recently…<br>Members of the public design and deliver their own research project	Academic research with citizen participation in collecting (not just responding) and / or analysing data<br>Third-sector or community organization's own research	Crowd-sourced science<br>Volunteer science<br>Community-led research (if using more recent meaning of citizen science)

Action Research	A form of co-production that is focused on making change in policy or society at large.	Supporting Youth Champions to influence policy at a national or regional level<br>Policy improvement programmes	Decision-making forum<br>Participative democracy
Scoping / feedback	Gathering views on a specific service directly from users of that service<br>Gathering information about public appetite for a new service<br>Limited to impact and quality improvements within the course / service / activity context	Student course evaluations<br>Post-event feedback form<br>Asking the public if they would be interested in X opportunity, in principle	Evaluation for effectiveness<br>Market research<br>Evaluation<br>Service evaluation<br>Quality improvement
Research participation	Taking part in a research project as an informed and consented participant	A clinical trial<br>Interview or focus group study<br>Online research survey	Public participation<br>*Note that some kinds of research studies may be called "evaluations"*

Narrative Example

It is easy for a research programme to move back and forth across these boundaries over time. For example, Rebecca and her team are planning a new clinical trial and to support the funding bid they start by asking patients in their diabetes clinic if they are satisfied with their current treatment and would be interested in trying something new (i.e. enrolling in a clinical trial). This is **scoping.** Incidentally, they notice that women tend to be much more dissatisfied with their treatment than men so they decide to do an analysis of treatment response in their clinic using routine data. This is **research**. Having published the work they decide to dig further into why there are gendered differences in treatment response and they work with a local group that supports patients with diabetes to design and deliver a new study that works for patients. This is **co-production**. In the meantime, they win the funding for their clinical trial and recruit a patient advisory group to help oversee the study. This is **public involvement**. They start recruiting patients through the clinic and testing their new treatment. This is **research participation.** Having completed both the trial and the gender-differences study, they host a public event to share their findings with doctors, nurses and patients more widely. This is **science communication**. A subset of the stakeholders, including individuals with diabetes, wish to develop further materials to highlight the findings and prompt discussion, and decide to **co-produce** a podcast. The group use the podcasts to inform a series of discussion events at a science festival to explore various facets of the situation: they gather **feedback** at the events from those who attended. Meanwhile, a gender-diversity community group decides they want to canvass their members' views, to go beyond the mostly binary gender classifications covered in the published research and they conduct a study with their members, taking some advice from Rebecca and her colleagues. This is **citizen science.** The local NHS Board decides to make changes to their public health approach to preventing diabetes and engage some patients as decision-makers about the new policy, and this process is followed by a student doing a Masters in Public Health. This is **action research**.

How we describe partners:

"Defining what you mean when talking about co-production also leaks over into the terminology used to refer to members of the public who are involved. I've had expert by experience, public contributor, lay member, service user, peer researcher, citizen + lived experience partner."

@SarahMarieOB

- A range of terminology is available and you should choose something carefully that fits with your focus and partners
- It is always a good idea to ask your partners how they would like to be addressed, and referred to in project paperwork
- "patient" may be appropriate when working in a clinical context with a specific group, but remember that
  a) We are all patients at some time or another – this term on its own is not highly descriptive
  b) It may be difficult to overcome power differentials between clinical academics and partners, if you stick with terminology like doctor and patient
- While we might talk about "community" or "lay" partners, meaning people who are not professional researchers or within academia, it is important to remember that colleagues within academia may also have relevant lived experience. In other words, don't make the mistake of implying that everyone from a particular group – e.g. people with an autism diagnosis, parents of a preterm born infant, diabetic people – bring a "lay" perspective. Some of them may also have relevant clinical, research or policy expertise for example.

Other resources:

- CMVM Public Engagement with Research web pages: https://www.ed.ac.uk/medicine-vet-medicine/engage-with-us/public-engagement-with-research
- National Co-ordinating Centre for Public Engagement: https://www.publicengagement.ac.uk/
- National institutes for Health Research: https://www.learningforinvolvement.org.uk/
- Co-Production Collective: https://www.coproductioncollective.co.uk/
- Knowledge Exchange Concordat: https://www.keconcordat.ac.uk/
- Sharing science through story: Fergus McAuliffe at TEDxDublin - YouTube: https://www.youtube.com/watch?v=cXJJvvjSB9c
- NIHR Reflective Questions: https://arc-w.nihr.ac.uk/Wordpress/wp-content/uploads/2020/05/Reflective-questions-Web-version-v1.2.pdf
- Imperial College PPI Resource Hub: https://www.imperial.ac.uk/patient-experience-research-centre/ppi/ppi-resource-hub/
- UKRI: Co-production in Research: https://www.ukri.org/about-us/policies-standards-and-data/good-research-resource-hub/research-co-production/
- UKRI: Knowledge Exchange Framework: https://kef.ac.uk/

Academic Papers

Farr, M., Davies, P., Andrews, H. et al (2021) Co-producing knowledge in health and social care research: reflections on the challenges and ways to enable more equal relationships. Humanit Soc Sci Commun 8, 105.

Fletcher-Watson, S., Brook, K., Hallett, S., Murray, F., & Crompton, C. J. (2021). Inclusive practices for neurodevelopmental research. *Current Developmental Disorders Reports*, *8*, 88-97

Redman, S., Greenhalgh, T., Adedokun, L., Staniszewska, S., Denegri, S., on behalf of the Co-production of Knowledge Collection Steering Committee (2021) Co-production of knowledge: the future. BMJ 2021;372:n434

Schroeder, D., Chatfield, K., Singh, M., Chennels, R., Herissone-Kelly, P., (2019). Equitable Research Partnerships. SpringerBriefs, UK

Staniszewska, S., Hickey, G., Coutts, P. et al. (2022) Co-production: a kind revolution. Res Involv Engagem 8, 4
