# Supplementary material for: Ethical considerations in public engagement: developing tools for assessing the boundaries of research and involvement
Source: Res Involv Engagem. 2024 Aug 7;10:83. doi: 10.1186/s40900-024-00617-8 (PMC11305016; doi:10.1186/s40900-024-00617-8)
Supplement: Supplementary file 2 — Supplementary Material 2 [file 40900_2024_617_MOESM2_ESM.pdf]

Ethical Public Engagement with Research

A self-assessment tool for judging whether your planned activity requires formal research ethics review or not, and for ethical conduct in PEwR

Professor Sue Fletcher-Watson
Co-Director of Research Ethics

Dr Iona Beange
Knowledge Exchange & Impact Officer

Professor Donald J. Davidson
Centre for Inflammation Research Academic Lead for Public Engagement

Jaime Garcia Iglesias
Mildred Baxter Postdoctoral Research Fellow

Dr Suzanne Goopy
Postgraduate-Taught Research Manager

Huayi Huang
Research Fellow, Usher Institute

Dr Fiona Murray
Public Engagement Coordinator

Carol Porteous
Patient Public Involvement Lead in Clinical Sciences

Dr Elizabeth Stevenson
Programme Director for MSc Science Communication and Public Engagement

Dr Sinead Rhodes
Senior Research Fellow

Faye Watson
Public Engagement with Research Manager

Introduction

This self-assessment tool was created by a working group, convened by the co-Directors of Research Ethics for the College of Medicine and Veterinary Medicine (CMVM). We aim to provide a more systematic way to evaluate whether a given academic activity, involving any non-academic partner (organisation or individual), requires formal evaluation by a research ethics committee. Formal research ethics involves a systematic process of analysis of risks and mitigation, and creation of a set of materials (e.g. study protocol, participant information sheet and consent form) to deliver the project. These are reviewed by members of a committee, to ensure the research planned is being conducted ethically.

While this process is essential for all research involving human participants, other research-linked activities do not necessarily require formal ethics review – please review our Taxonomy[1] document for examples. In fact, it can undermine the power placed in the hands of non-academic partners if they are treated as “subjects” of the research rather than expert partners, or public consumers.

Individual researchers and Research Ethics Committee members can use this tool to evaluate the need for a formal ethical review, and refer to it when making enquiries to committees regarding potential review. Ultimately research ethics committees reserve the right to request full ethical paperwork be prepared and submitted if in any doubt. We aim to be proportionate, and respect the need for public engagement with research including clinical trial advisory boards, priority-setting exercises, co-production of new tools and outputs etc. However, we must also always protect the rights and wellbeing of community members, especially those who might be considered vulnerable. In addition, we have to consider reputational risk to the University of Edinburgh and to our staff and students.

What is Research? What is Public Engagement with Research?

Research is conceived broadly to include staff and student research activities to derive generalizable or transferable new knowledge with appropriate rigorous and robust methods (including pilot, unfunded, funded, and commissioned research). It also may include some consultancy, and knowledge exchange and impact activities, including public engagement with research.

Public engagement with research (PEwR) describes the many ways we can share research with new audiences and listen in response. It is a two-way process that enables change and has mutual benefit. High-quality engagement can enhance the impact of research. Engagement methods are influenced by the stage and nature of research activity. There are many ways we can use public engagement with research; to reveal knowledge, to inspire interest, to listen to new perspectives, to consult people with lived experience, to build trust in new technologies, to collaborate and to co-create research.

Getting Help

When using this tool we recommend you:

- Talk to your supervisor, line manager, and academic collaborators
- Discuss your plans with any external partners already linked to your project
- Review our taxonomy of research terms[1]
- Approach the College Public Engagement with Research Manager for advice[2]
- Contact emrec@ed.ac.uk or your usual ethics committee if you are still not sure if formal ethics review is needed

Using This Tool

1. Have a clear sense of what your planned activity will involve, though it might be a good idea to review this tool relatively early in the planning stage. You might want to make adjustments to your plans following your first pass through the self-assessment
2. Review each of the four number sections below, determining to what extent your planned activity is more or less likely to need formal research ethics review
   a. In each section, the first table (in orange) has the key determinants which will help to categorise an activity as needing / not needing ethics review
   b. The second table (in blue) in each section adds additional things to consider
3. Check your self-assessment outcome with a suitably expert colleague, such as the College Public Engagement with Research Manager
4. If you think part or all your project needs research ethics review, contact EMREC or your usual research ethics committee to confirm, and proceed to follow their ethics review process[3]
5. If you are confident your project doesn't need research ethics review, move on to part 2 of the tool which is designed to facilitate ethical conduct in public engagement with research

Considerations when Determining whether Formal Research Ethics Review is Needed.

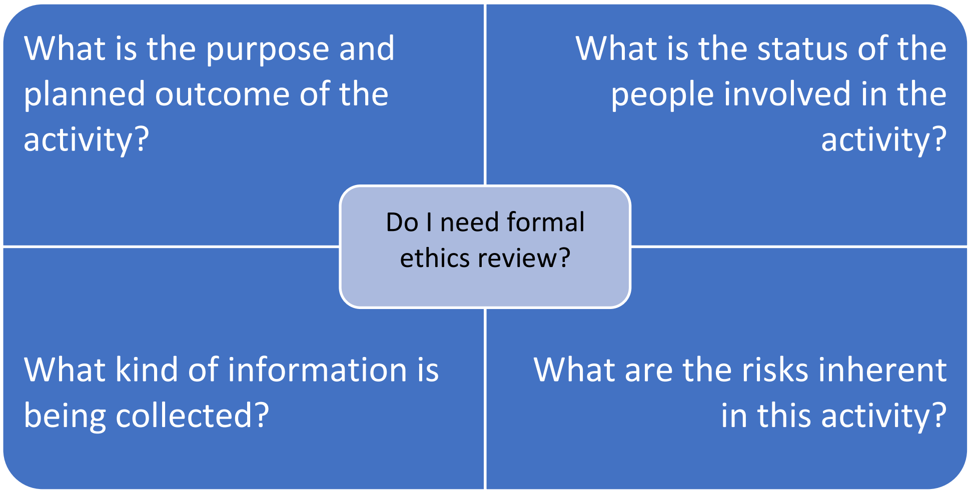

Please proceed to the next two pages for the decision-making tool

---

[1] https://uoe.sharepoint.com/sites/EMREC/SitePages/Public-Engagement-with-Research.aspx
[2] https://www.ed.ac.uk/medicine-vet-medicine/about/contacts/communications-marketing-engagement
[3] https://uoe.sharepoint.com/sites/EMREC

1. What is the purpose and planned outcome of the activity?

Probably doesn't need formal ethics review	Almost certainly needs formal ethics review
To plan a new project or activity	To answer a specific research question
Main goal is relationship-building and insight	Main goal is systematic generation of knowledge
Activity is designed to share research findings	Activity is designed to generate research findings

Less likely to need formal ethics review	More likely to need formal ethics review
To develop a list of research questions or priorities	To formally synthesise and describe attitudes to, opinions on, or experience of research or practice
Information gathered will be used to help write a grant or to co-create public-facing materials such as a web page, flyer or video	Information gathered will be used to write a journal paper
Perspectives will inform delivery of an event, activity or project	Perspectives will inform the wider research literature / general understanding
Information will be used to directly inform the design and delivery of an activity or project	Information will be formally analysed in order to answer a research question
Activity is part of the early conceptualisation phase of an activity or project	Activity follows from a phase of research study design

2. What is the status of the people involved in the activity?

Probably doesn't need formal ethics review	Almost certainly needs formal ethics review
They will be advisors to the team	They will be contributing data to the team
They will be contracted (formally or informally) consultants on the project	They will be signing consent forms to be part of the project

Less likely to need research ethics review	More likely to need research ethics review
They will be equal partners with the academic team	They will be undertaking tasks determined by academics
They will not be required to share personal details or report on their experiences, though their contributions will be informed by those experiences	They will be required to share personal details for sample description and analysis
They will be personally invited, or self-refer to the role	They will be recruited to the role (potentially in large numbers)
They will often be community leaders, activists, and associated with organised groups	They will be anyone who meets a defined set of inclusion criteria
They may have ongoing buy-in to the activity	Their involvement stops once data have been contributed
They may personally benefit, e.g. via learning new skills or making new connections	They are unlikely to personally benefit from the research, except in the form of payment for their time

3. What kind of information is being collected and what will happen to it?

Probably doesn't need formal ethics review	Almost certainly needs formal ethics review
Information may be organised , e.g. using filenames, labelling and colour coding	Information will be formally analysed
Information cannot and will not be re-used for future projects	Information can and may be re-used for future projects

Less likely to need research ethics review	More likely to need research ethics review
Asking about expert opinions on a topic	Asking about personal experiences
Collecting feedback on an event (and using this to inform future, similar events)	Collecting information about attitudes or opinions and using this to draw generalised conclusions
Sessions will be minuted or notes taken	Sessions will be recorded and transcribed
People involved will not provide personal details (except contact details)	People involved will provide personal details (as well as contact details)
Information collected will be deleted soon after the activity	Information collected will be kept for months or longer
Information will not be archived long-term	Information will be archived long-term

4. What are the risks inherent in your study?

Probably doesn't need formal ethics review	Almost certainly needs formal ethics review
People involved will be active and engaged	People involved will be passive and controlled
People involved will be free to change how they participate without withdrawing from the study	People involved have a choice between following protocol or withdrawing from the study

Less likely to need research ethics review	More likely to need research ethics review
People will be involved in decision-making	People will be told what to do by others
Ideas for activities are generated by the group of involved people	What happens is pre-determined by the academic team and following a protocol
People will take part in routine activities, such as meetings or as an audience	People will try something novel or experimental
People will be provided with training and materials to help them understand the study context in full	People will be provided with information only pertaining to their specific role
People will be credited for their role, in a manner of their choosing	People's involvement will and must be anonymised fully

Please proceed to the next page for guidance on ethical conduct of public engagement activities, without formal ethics review

Managing the risks involved in public engagement with research

The guidance below provides a reflective framework to help plan public engagement with research (including co-production, science communication and more) in an ethical manner. We recommend that you review this list as a team, and / or get a second opinion before proceeding.

Data minimisation and security

- If you are collecting personal info (e.g. contact details) complete a Data Protection Impact Assessment (DPIA)[4]
- Only use information for the purpose for which it was collected
- Minimise the amount of data you collect at all times: e.g. take notes instead of audio recording
- Make sure everyone is clear what information is being collected and for what purpose
- Securely delete raw information (e.g. handwritten notes) as soon as it is no longer in use
- Hold information (e.g. contact details) in a secure location just as you would with research data
- Establish confidentiality rules at events or meetings, perhaps using the Chatham House Rule[5]

Safeguarding Collaborators and Emotional Labour

- Carefully consider the potential for distress to your community partners: at a minimum, get a second opinion from colleagues with suitable insight and expertise
- Put measures in place to prevent harm and minimise distress. Have a plan for responding to distress should it arise, such as a safe and quiet breakout space to retreat to.
- Put mitigations in place such as content warnings and freely share information about reputable support organisations (e.g. free helplines provided by quality third sector organisations)
- Plan for what you will do if community partners taking part in the activity share more personal information than you anticipated.

Professional Conduct and Intellectual Property

- Provide absolute clarity on what is expected of community partners and how their contribution will be used
- Consider co-producing a Contract of Engagement or Terms of Reference for the group, to make rights and responsibilities explicit and clear
- Do not over-promise the influence they will have on the specific project, or wider field
- Ensure community partners understand their rights in relation to any intellectual property resulting from the shared activity.
- If significant intellectual property may be generated, consider a written agreement up front or at a suitable point during the collaborative process.
- Feed back to community partners about what has changed as a result of their involvement

Power Imbalances

- Make sure you understand and recognise the likely power imbalance between you, as an academic, and your community partners
- Pay extra attention to power imbalances when working in a cultural context where you are an outsider
- Work to minimise this imbalance, e.g. in your choice of location, in the materials you share
- Ensure community partners are given all the information they need to effectively contribute to the project
- Respect what community partners want to tell you even if it is not what you were hoping to hear
- Cultivate emancipatory opportunities wherever possible, e.g. by offering co-authorship on journal articles or study reports, while recognising that the opportunities that community partners value may be different to those you consider valuable

---

[4] https://www.ed.ac.uk/data-protection/data-protection-impact-assessments
[5] https://www.chathamhouse.org/about-us/chatham-house-rule
